# Multispecies Adulteration Detection of Camellia Oil by Chemical Markers

**DOI:** 10.3390/molecules23020241

**Published:** 2018-01-25

**Authors:** Xinjing Dou, Jin Mao, Liangxiao Zhang, Huali Xie, Lin Chen, Li Yu, Fei Ma, Xiupin Wang, Qi Zhang, Peiwu Li

**Affiliations:** 1 Oil Crops Research Institute, Chinese Academy of Agricultural Sciences, Wuhan 430062, China; douxj521@163.com (X.D.); maojin106@whu.edu.cn (J.M.); xiehuali151163@126.com (H.X.); chenlinhcl@163.com (L.C.); yuli0201010133@gmail.com (L.Y.); mafeicpu@163.com (F.M.); wangxiupin@caas.cn (X.W.); zhangqi521x@126.com(Q.Z.); 2 Key Laboratory of Biology and Genetic Improvement of Oil Crops, Ministry of Agriculture, Wuhan 430062, China; 3 Laboratory of Quality and Safety Risk Assessment for Oilseed Products (Wuhan), Ministry of Agriculture, Wuhan 430062, China; 4 Key Laboratory of Detection for Mycotoxins, Ministry of Agriculture, Wuhan 430062, China; 5 Quality Inspection and Test Center for Oilseed Products, Ministry of Agriculture, Wuhan 430062, China; 6 Hubei Collaborative Innovation Center for Green Transformation of Bio-Resources, Wuhan 430062, China

**Keywords:** multispecies-adulteration, characteristic markers, camellia oil, solid phase extraction

## Abstract

Adulteration of edible oils has attracted attention from more researchers and consumers in recent years. Complex multispecies adulteration is a commonly used strategy to mask the traditional adulteration detection methods. Most of the researchers were only concerned about single targeted adulterants, however, it was difficult to identify complex multispecies adulteration or untargeted adulterants. To detect adulteration of edible oil, identification of characteristic markers of adulterants was proposed to be an effective method, which could provide a solution for multispecies adulteration detection. In this study, a simple method of multispecies adulteration detection for camellia oil (adulterated with soybean oil, peanut oil, rapeseed oil) was developed by quantifying chemical markers including four isoflavones, trans-resveratrol and sinapic acid, which used liquid chromatography tandem mass spectrometry (LC-MS/MS) combined with solid phase extraction (SPE). In commercial camellia oil, only two of them were detected of daidzin with the average content of 0.06 ng/g while other markers were absent. The developed method was highly sensitive as the limits of detection (LODs) ranged from 0.02 ng/mL to 0.16 ng/mL and the mean recoveries ranged from 79.7% to 113.5%, indicating that this method was reliable to detect potential characteristic markers in edible oils. Six target compounds for pure camellia oils, soybean oils, peanut oils and rapeseed oils had been analyzed to get the results. The validation results indicated that this simple and rapid method was successfully employed to determine multispecies adulteration of camellia oil adulterated with soybean, peanut and rapeseed oils.

## 1. Introduction

Food fraud, motivated by financial benefits, is a common phenomenon around the world, especially for oils, dairy products, fruit juices, honey, wine and seafood [1,2]. Among the food mentioned before, oil adulteration accounts for a large proportion and attracts serious attention from researchers [2,3,4]. Edible oil plays an indispensable role in our daily life as the sources of essential fatty acids, carotenoids, and lipid-soluble vitamins like vitamin E and vitamin K [5,6,7]. Besides, unknown adulteration of oils without clear labels may lead to allergy, which affects human health. Hence, an effective method for discriminating the adulteration of oils needs to be established urgently [3].

Camellia oil, a kind of vegetable oil that possesses high nutritional value, is popular to consumers for its similar fatty acid composition to olive oil. It is therefore called the “eastern olive oil”. Camellia oil is rich in fat-soluble vitamins and other monounsaturated fatty acids, especially oleic acid accounting for more than 75% of total fatty acid contents and other nutrients [8]. Previous study has summarized the effects of antioxidant activity in camellia oil for human health including preventing cardiovascular cirrhosis, lowering the blood pressure, and reducing blood fat [9,10]. Compared with soybean oil, rapeseed oil and peanut oil, the special camellia oil is usually expensive in China. Therefore, camellia oil has a high risk of economically motivated adulteration.

Various analytical methods were proposed and used to detect adulteration of vegetable oils. Many researchers developed a series of methods for rapid determination of oil adulteration, which depended on magnetic, optical, or electrical signals of entire samples. These methods include nuclear magnetic resonance (NMR) [11], near infrared spectroscopy (NIR) [12], mid-infrared spectrum (MIR) [13], Raman spectrometry [14], electronic nose [15] and ion mobility spectrometry [16,17,18]. The analytical methods above are usually coupled with chemometric methods and applied to the evaluation of the quality of edible vegetable oils. Targeted edible vegetable oils were described by magnetic, optical, or electrical signal to acquire a large number of data samples to construct a prediction model. Meanwhile, other methods were developed based on a set of chemical components. Chromatographic techniques such as GC, capillary electrophoresis (CE) and LC were used to identify adulteration based on the triglyceride (TAG), fatty acids (FAs) and sterols in edible oils [3,4,19,20,21,22]. However, the probability of authenticity of oils could be predicted by chemometric models. In practice, adulteration is deliberately conducted with various types of oils. Therefore, it is one of the biggest challenges to identify multispecies adulteration in expensive oils. Furthermore, model based authentication methods could not provide confirmed results for forensic evidence.

Compared with the detection methods above, the detection of characteristic chemical markers in vegetable oils has attracted increasing attention. Obviously, DNA was the first candidate as a marker of vegetable oils. Previous studies showed that DNA could be a marker for authentication or traceability of extra virgin olive oil [23,24]. Since the concentration of adulterated oil’s DNA would decrease significantly or even disappear after deep refining, it is not precise to detect adulteration by DNA based method. Besides, small-molecule markers were used to detect adulteration. Tocopherols and tocotrienols were proven to be the markers for detecting adulteration of palm and grapeseed oils adulterated in other oils, providing that high amounts of tocopherols and tocotrienols in palm and grapeseed oils can be detected [25]. Another example was that 4, 4′-dimethylsterols in hazelnut oil were proven to be markers for the detection of adulteration with hazelnut oil in olive oil [26]. Compared with the rapid detection methods, the characteristic chemical marker based adulteration detection method could provide more specific and confirmed conclusions. For an unknown sample, however, an exclusive method should be used to determine whether the adulterant exists or not by detecting the chemical marker of this adulterant. Therefore, it is necessary to develop the simultaneous analytical method of chemical markers in several cheap oils for multispecies adulteration detection.

According to the previous reports, four isoflavones were simultaneously found in soybean oil, while resveratrol was only in peanut oil. Therefore, four isoflavones and resveratrol could be taken as characteristic markers of soybean oil and peanut oil, respectively [27,28]. Meanwhile, sinapic acid was selected as a marker for rapeseed oil as its significant higher content in rapeseed oil than other edible oils. In this study, we investigated whether four isoflavones (daidzein, genistein, daidzin and genistin), sinapic acid and resveratrol could be taken as the chemical markers for soybean oil, rapeseed oil and peanut oil, respectively. Besides, the other aim of this study was to develop a simple method to identify multispecies adulteration in camellia oil by detecting characteristic markers of inexpensive vegetable oils (soybean, peanut and rapeseed oils) simultaneously using SPE-LC-MS/MS.

## 2. Results and Discussion

### 2.1. Method Validation

The method was validated using the following parameters: linearity, limit of detection (LOD), limit of quantification (LOQ), analytical recovery and LOD and LOQ for LC-MS/MS system. Calibration curves were established and evaluated by injecting the standards dissolved in methanol. Calibration curves were prepared at the concentration levels of 0.1, 0.5, 1, 5, 10, 20, 50, 100, 200, 500, 1000 and 2000 ng/mL for mixed standards. As the previous literature [29] reported, the LOD was calculated as the lower concentration with acceptable chromatography and the presence of all transitions with the signal-to-noise ratio of at least 3, while the LOQ was the lowest concentration that met the LOD criteria with a signal-to-noise ratio of at least 10. The results indicated that the LODs and LOQs of the target compounds ranged from 0.05 to 0.16 ng/mL and from 0.06 to 0.53 ng/mL, respectively. The calibration equations of all of the compounds were linear with the correlation coefficient (*R*^2^) greater than 0.9955, indicating that good linearity could be obtained within a wide range. The detailed information including the LOD, LOQ, linear range and equations of the target compounds was given in Table 1.

Accuracy studies were performed to evaluate the method performance, which was estimated from three batches of samples in triplicate, as the previous literature described [30]. Accuracy was evaluated by the percentage of the recovery by using the spiked samples at different concentrations. The recovery was calculated by difference values between the blank oil and spiked oil. As illustrated in Table 2, three levels (10 ng/g, 50 ng/g and 250 ng/g) of the standards were added into peanut oils and the mean recoveries for the target compounds ranged from 79.7% to 113.5%, which could be acceptable for analysis. Besides, the standard deviations (SDs) of the parallel assays were mostly less than 10%, indicating good repeatability for the extraction method. Precisions including within-day variance and between-day variance were evaluated by analyzing the spiked samples at different concentration levels. The intra-day precision expressed by the relative standard deviation (RSD, %) was obtained through the analysis of the same sample for three times under the same condition during a day while the inter-day precision was tested on five consecutive days. As presented in Table 2, the intra-day precisions for the target compounds ranged from 0.61% to 7.37% while the inter-day precisions ranged from 2.88% to 11.91%, suggesting an acceptable variance for analysis.

### 2.2. Simultaneous Detection of Multispecies Adulteration of Camellia Oil

**Figure 1:**
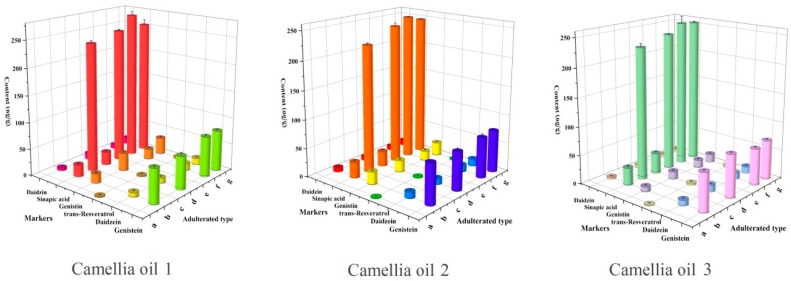
Concentration of the target markers after adulteration (*n* = 3): (a) camellia oil adulterated with 10% peanut oil; (b) camellia oil adulterated with 10% soybean oil; (c) camellia oil adulterated with 10% rapeseed oil; (d) camellia oil adulterated with 10% peanut oil and 10% soybean oil; (e) camellia oil adulterated with 10% peanut oil and 10% rapeseed oil; (f) camellia oil adulterated with 10% soybean oil and 10% rapeseed oil; and (g) camellia oil adulterated with 10% soybean oil 10% rapeseed oil and 10% peanut oil.

**Figure 2:**
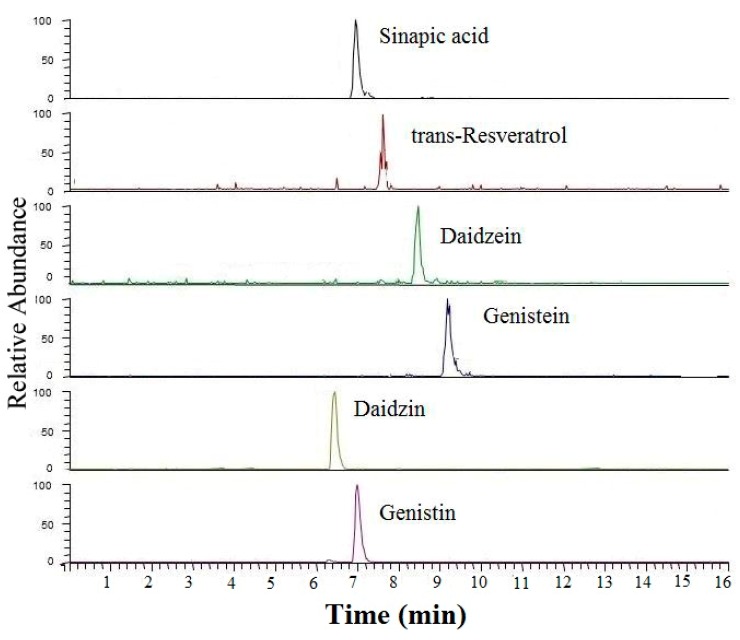
Chromatographic peaks of characteristic markers in adulterated camellia oil, which was adulterated with 10% soybean oil, 10% peanut oil and 10% rapeseed oil.

The detection of multispecies adulteration in camellia oil was conducted as an example in this work. In the beginning, potential target markers of soybean oils, peanut oils and rapeseed oils were determined with the developed method mentioned above. The summarized and detailed quantification results for markers in four kinds of edible oils were presented in Tables S1 and S2. As is shown in Table S1, four isoflavones were simultaneously detected in soybean oils, among which genistein, genistin and daidzein were present only in soybean oils and therefore could be used as chemical markers of soybean oil. Meanwhile, trans-resveratrol was employed to be markers of peanut oils. Although sinapic acid was also detected in rapeseed oils and soybean oils, it is not found in camellia oil and therefore could be taken as a chemical marker for detecting the adulteration of rapeseed or soybean oil. To test the effectiveness of this method, three camellia oils were selected as the original authentic oils. According to the previous studies, adulteration level of 10% was used to evaluate the detection method [31]. Therefore, the adulteration level was set to 10%, while quality ratio was used for binary adulteration and ternary adulteration. The unispecies adulteration with peanut oil (a); soybean oil (b) and rapeseed oil (c); bispecies adulteration with peanut oil and soybean oil (d); peanut oil and rapeseed oil (e); soybean oil and rapeseed oil (f); and ternary adulterated with peanut oil, soybean oil and rapeseed oil (g) were prepared, respectively. Then, target markers were detected by the developed method. The contents of markers in these adulterated oils were illustrated in Figure 1. As displayed in Figure 1, the results suggested that four isoflavones simultaneously appeared only in adulterated oil with soybean oil (b, d, f and g). Trans-Resveratrol could be detected only in adulterated oil with peanut oil. Sinapic acid could be taken as the marker of rapeseed oil because of the specific distribution and significantly higher content in rapeseed oils than other edible oils. As shown in Figure 1, the contents of sinapic acid in camellia oils adulterated with 10% rapeseed oil ranged from 200 to 300, which was about 100 times higher than that in soybean oil. Therefore, when two or three out of soybean oil, peanut oil and rapeseed oil were added into camellia oil, the distributions of the chemical markers conformed to the principle we described previously. In this way, we could conclude that the oil was adulterated with soybean oil if four isoflavones appeared at the same time. In a similar way, the adulteration with peanut oil or rapeseed oil could be determined if trans-resveratrol or a high content of sinapic acid was detected. Detailed data of Figure 1 was supplied in Table S3. Besides, the more intuitive result was shown in Figure 2, distinct chromatographic peaks of isoflavones, trans-resveratrol or sinapic acid appeared when camellia oil was adulterated with 10% soybean oil, 10% peanut oil and 10% rapeseed oil.

## 3. Experimental

### 3.1. Reagents and Materials

Methanol, *n*-hexane and acetic acid of HPLC grade were purchased from Sinopharm Chemical Reagent Co., Ltd. (Shanghai, China). Ultra-pure water was obtained from a Milli-Q water purification apparatus (Millipore Co., Ltd., Milford, MA, USA). SPE cartridges bond with diol (500 mg, 6 cc) were purchased from water.

### 3.2. Standards

All standards were supplied by Sigma-Aldrich Co., Ltd. (Shanghai, China). Detailed information was as follows: daidzein (≥98%, synthetic (Sigma)), daidzin (≥95.0% (HPLC), (Sigma)), genistein (synthetic, ≥98% (HPLC), powder (Sigma)), genistin (from Glycine max (soybean), ≥95% (HPLC), (Sigma)), trans-resveratrol (≥99% (HPLC)) and sinapic acid (analytical standard (Sigma-Aldrich)). All standards (10.0 ± 0.1 mg) were accurately weighed into a 10 mL volumetric flask and then separately dissolved with methanol as the stock solutions (1 mg/mL). A series of standard solutions were prepared by diluting the stock solutions with methanol. To ensure constant concentrations of standard solutions, all of the standard solutions were transferred into a brown volumetric flask with screw cap, sealed with parafilm, and then stored in the dark at −20 °C in a refrigerator until use.

### 3.3. Sample Preparation

To ensure the authenticity of the oils used in this study, camellia seeds, soybeans, peanuts and rapeseeds were gathered from supermarkets. They were stored at 20 °C in darkness until use. The oilseeds were put into an oil presser after being shelled one by one. The oil presser was cleaned after treating each sample to avoid cross-contamination. The oils were placed into a 50 mL centrifuge tube with cap respectively and then centrifuged at 5000 rpm for 10 min. The supernatant liquid was transferred into a brown volumetric flask for reservation. The adulterated camellia oils were prepared by adding soybean, peanut or rapeseed oil squeezed freshly according to quality ratio (m/m). Then, the mixture was vortexed and ultrasonic to obtain a high mixing homogeneity. All of the oil samples were stored in a room at 20 °C before use.

To validate this method, peanut oil samples were spiked with standard mixtures of daidzein, daidzin, genistein, genistin, resveratrol and sinapic acid at different concentration levels of 10 ng/g, 50 ng/g and 250 ng/g. Then, methanol remaining in the oils was evaporated under nitrogen gas before the next step.

### 3.4. SPE Procedures

Since the selected chemical markers were present at a trace level in the oil matrix, SPE with diol cartridges was used for purification and concentration according to the previously published literature [32,33,34].

First, SPE cartridges were sequentially preconditioned with 5 mL methanol and 5 mL n-hexane. One gram of oil sample was accurately weighed into a 10 mL centrifuge tube. Then, the oil was diluted with 8 mL *n*-hexane and vortex-shaken for 30 s to ensure well dispersion. The sample solution was loaded to the cartridge by gravity, and, consequently, the solvent went through, leaving target components on the cartridge. Subsequently, the cartridge was washed with 5 mL *n*-hexane to remove nonpolar fractions of the oil. Finally, the target compounds remaining on the cartridge were eluted by passing through 2 × 3 mL methanol, which were recovered in a 10 mL centrifuge tube. Then, the elution liquid was evaporated to dryness under a gentle stream of nitrogen gas. The residue was dissolved with 0.5 mL methanol and vortex-shaken for 1 min to ensure that the target compounds were dissolved entirely. The final extracts were filtered through 0.45 µm organic filter before being injected into LC-MS/MS.

### 3.5. LC-MS/MS Conditions

Analyses were carried out on an Accela HPLC system coupled to Thermo TSQ Quantum Ultra EMR (Thermo Fisher Scientific, Waltham, MA, USA).

Chromatographic separation was performed using a C_18_ column (Hypersil Gold, 100 mm × 2.1 mm i.d., 3 µm, Thermo Fisher Scientific, Waltham, MA, USA) at 30 °C. The oven temperature was set at 30 °C, and gradient elution was performed with a binary solvent system consisting of 0.01% acetic acid in methanol (mobile phase A, *v*/*v*) and 0.01% acetic acid in water (mobile phase B, *v*/*v*) at a flow rate of 0.2 mL/min. The gradient elution program was applied as follows: 10% A for 2 min, 10–30% A over 0.5 min, 30–55% A over 2.5 min, 55–70% A over 3 min, 70–85% A over 5 min, and finally returned to the initial condition over 0.5 min and held for 2.5 min to equilibrate the column before the next injection. The total analysis time was 16 min, and the injection volume was 3 µL for a good peak profile.

Mass spectrometry equipped with electrospray ionization (ESI) source was performed in negative and positive selected reaction monitoring (SRM) modes. The spray voltage was set to −4.0 kV for negative scan mode and 3.5 kV for positive scan mode. The capillary temperature was 330 °C, sheath gas pressure (N_2_) was 35 units, auxiliary gas pressure (N_2_) was 5 units, collision gas (Ar) was 1.0 mTorr, and scan time was 0.1 s.

The mass spectrometer was tuned in both positive- and negative-ionization ESI modes for individual standards. Generally, the signal intensity and signal-to-noise ratio for most compounds in positive-ionization mode were both much greater than in negative-ionization mode. As expected, alcohols and carboxylic acids like sinapic acid and trans-resveratrol performed higher responses in negative-ionization mode. To obtain more sensitive abundance for target compounds, mass spectrometric parameters like the monitoring precursor ion, product ion, tube lens and collision energy were optimized by infusion of each individual analyte (1000 ng/mL in methanol) at 20 µL/min. The specific parameters were displayed in Table 3.

### 3.6. Data Processing

Peak identification was required for accurate qualitative analysis of target compounds. The peaks of oil sample extracts were identified by comparing the retention time (RT) as well as monitoring ion pairs with specific standard solutions. The identification criteria included RT within 0.2 min of the average calibrator RT, presence of two transitions and relative ion intensities (% of base peak) within 20% [35], and the quantification was achieved by an external standard method. Different levels of standard solutions, which were prepared by diluting the stock solutions with methanol, fitted the standard curve by the relationship between the concentration and peak area. To achieve high sensitivity, the fragments showing the highest abundance were selected as the monitoring ion pairs. Data acquisition and processing were carried out by using the Xcalibur software version 2.0.7 (Thermo Fisher Scientific, Waltham, MA, USA).

## 4. Conclusions

A simple method for detecting several markers in soybean oil, peanut oil and rapeseed oil was applied to discriminate potential adulteration of camellia oil after validation. After analyzing the characteristic markers in authentic camellia oil, we found that most of the markers were absent except trace daidzin. Therefore, the analysis of these markers in camellia oil can be used for identification of the adulterants if there is one or several of them including soybean oil, rapeseed oil and peanut oil. As expected, discrimination of adulteration in camellia oil including single or multispecies adulterants was achieved successfully by a characteristic marker or specific distributions, and the effectiveness of this method was verified. Moreover, compared with the previous studies in Table 4, the developed method might detect multispecies adulteration simultaneously. Hence, the present method may be routinely used for detection of targeted soybean oil, peanut oil and rapeseed oil in camellia oil. In addition, another advantage of this method was that identification of potential adulteration for soybean oil, peanut oil and rapeseed oil could be achieved. However, it could be more complex and unpredictable when various kinds of oils were blended into expensive oil. In addition, for this method, further studies would be necessary to find more markers of other oils. Meanwhile, the distributions of these markers might be different in the same species of oils due to different origins or climates, and therefore high sensitivity of a determination method was needed.

## Figures and Tables

**Table 1:** Standard curve, limit of detection and limit of quantification.

Compounds	Linear Range (ng/mL)	Linear Equation	Correlation Coefficients	LOD (ng/mL)	LOQ (ng/mL)
Daidzin	0.16–2000	*Y* = −38155.2 + 47814.1 × *X*	0.9999	0.05	0.16
Sinapic acid	0.45–2000	*Y* = −8606.82 + 1266.41 × *X*	0.9977	0.14	0.45
Genistin	0.06–2000	*Y* = 635.271 + 28034.5 × *X*	0.9996	0.02	0.06
trans-Resveratrol	0.53–1000	*Y* = −3526.2 + 910.121 × *X*	0.9965	0.16	0.53
Daidzein	0.21–2000	*Y* = 16883 + 14110.6 × *X*	0.9985	0.06	0.21
Genistein	0.21–2000	*Y* = 5674.39 + 6132.46 × *X*	0.9955	0.07	0.23

LOD: limit of detection; LOQ: limit of quantification.

**Table 2:** Validation of the analytical method including precision and accuracy.

Compounds	Intra-Day Precision			Inter-Day Precision			Accuracy, Recovery		
	(%, RSD, *n* = 3)			(%, RSD, *n* = 5)			(%, mean ± SD, *n* = 3)		
	10 ng/g	50 ng/g	250 ng/g	10 ng/g	50 ng/g	250 ng/g	10 ng/g	50 ng/g	250 ng/g
Daidzin	1.51	1.5	0.9	10.14	4.39	5.23	96.5 ± 1.1	99.6 ± 1.7	86.9 ± 6.0
Sinapic acid	7.21	3.36	1.45	2.88	7.72	11.52	110.2 ± 3.3	101.5 ± 4.0	113.5 ± 6.9
Genistin	3.37	0.95	2.23	11.91	5.96	6.15	79.7 ± 2.9	88.8 ± 3.5	81.7 ± 4.5
trans-Resveratrol	7.37	4.24	3.36	11.58	7.56	9.55	85.2 ± 14.9	80.4 ± 3.0	84.1 ± 2.3
Daidzein	1.91	0.61	0.87	5.48	5.13	5.29	92.9 ± 2.0	96.4 ± 3.8	87.0 ± 2.2
Genistein	3.7	0.87	4.49	12.3	7.97	5.52	103.5 ± 3.9	97.8 ± 1.0	86.1 ± 3.7

**Table 3:** LC-MS/MS parameters of the target compounds.

Compound	Scan Mode	Retention Time (min)	Parention (*m*/*z*)	Production (*m*/*z*)	Collision Energy (eV)	Tube Lens (V)
Daidzin	+	6.43	417	199/255	45/22	160
Sinapic acid	−	6.96	223	193/208	25/17	136
Genistin	+	7.01	433	271	27	127
trans-Resveratrol	−	7.57	227	143/185	20/22	137
Daidzein	+	8.41	255	137/199	26/30	132
Genistein	+	9.18	271	153/215	27/25	127

**Table 4:** Comparison of adulteration detection method with the previous studies.

Authentic Oil	Adulterant	Chemical Markers	Adulteration Type	Detection Technique	Reference
Olive oil	Almond oil	Lupeol α-amyrin	Bispecies adulteration	GC or GC-MS	[36]
	Hazelnut oil				
Olive oil	Sunflower	Δ^7^-Stigmastenol and campesterol	Bispecies adulteration	GC or GC-MS	[37]
	Soybean oil				
Olive oil	Rapeseed oil	Brassicasterol	Unispecies adulteration	GC or GC-MS	[37]
Olive oil	Sunflower oil	Trigonelline Carnitine/acylcarnitines	Multispecies adulteration	CE-MS/MS	[38]
	Corn oil				
	Soybean oil				
Olive oil	Hazelnut oil	(*E*)-5-methylhept-2-en-4-one	Unispecies adulteration	RPLC-GC	[39]
Sesame oil	Refined corn oil	2-Propenal	Bispecies adulteration	HS-SPME GC-TOFMS	[40]
	Refined soybean oil				
Camellia oil	Soybean oil	Daidzin	Multispecies adulteration	HPLC-MS/MS	This work
		Daidzein			
	Peanut oil	Genistein			
		Genistin			
	Rapeseed oil	trans-Resveratrol			
		Sinapic acid			

CE: capillary electrophoresis; RPLC: reversed phase liquid chromatography; HS-SPME: Headspace Solid phase Micro Extraction; TOFMS: Time of Flight mass spectrometry.

## Supplementary Materials

The supplementary tables are available online.
