# Supplementary material for: Multispecies Adulteration Detection of Camellia Oil by Chemical Markers
Source: Molecules. 2018 Jan 25;23(2):241. doi: 10.3390/molecules23020241 (PMC6017810; doi:10.3390/molecules23020241)
Supplement: Supplementary file 1 [file molecules-23-00241-s001.pdf]

Supplementary Materials

Table S1. Summarized contents of target markers in four kinds of edible oils.

Table S2. Detailed results for the determination of target markers in four kinds of edible oils.

Table S3. Concentration of the target markers after adulteration.

Table S1. Contents of target markers in four kinds of edible oils

Sample \ Analysts	Daidzin ng/g			Genistein ng/g			Genistin ng/g			Daidzein ng/g			trans-Resveratrol ng/g			Sinapic acid ng/g		
	Mean	Min	Max	Mean	Min	Max	Mean	Min	Max	Mean	Min	Max	Mean	Min	Max	Mean	Min	Max
Camellia oil (n = 12)	0.06 ± 0.00 [a]	ND [b]	0.46		ND			ND			ND			ND			ND	
Peanut oil (n = 7)		ND			ND			ND			ND		31.76 ± 30.56	7.06	87.49		ND	
Soybean oil (n = 10)	5.89 ± 5.74	1.09	19.85	137.0 ± 143.0	13.81	467.29	347.3 ± 239.9	6.15	643.25	154.4 ± 140.4	16.92	492.29		ND		3.31 ± 5.95	0.45	20.10
Rapeseed oil (n = 9)		ND			ND			ND			ND			ND		2088.3 ± 1038.3	1383.87	4782.28

[a] Average value ± Standard Deviation; [b] ND, below the limit of detection (LOD).

Table S2. Detailed results for the determination of target markers in four kinds of edible oils.

Sample ng/g	Daidzin	Sinapic acid	Genistin	trans-Resveratrol	Daidzein	Genistein
Camellia oil 1	ND	ND	ND	ND	ND	ND
Camellia oil 2	0.46	ND	ND	ND	ND	ND
Camellia oil 3	ND	ND	ND	ND	ND	ND
Camellia oil 4	ND	ND	ND	ND	ND	ND
Camellia oil 5	ND	ND	ND	ND	ND	ND
Camellia oil 6	ND	ND	ND	ND	ND	ND
Camellia oil 7	ND	ND	ND	ND	ND	ND
Camellia oil 8	ND	ND	ND	ND	ND	ND
Camellia oil 9	0.1	ND	ND	ND	ND	ND
Camellia oil 10	ND	ND	ND	ND	ND	ND
Camellia oil 11	ND	ND	ND	ND	ND	ND
Camellia oil 12	ND	ND	ND	ND	ND	ND
Peanut oil 1	ND	ND	ND	21.21	ND	ND
Peanut oil 2	ND	ND	ND	87.49	ND	ND
Peanut oil 3	ND	ND	ND	15.35	ND	ND
Peanut oil 4	ND	ND	ND	18.31	ND	ND
Peanut oil 5	ND	ND	ND	7.06	ND	ND
Peanut oil 6	ND	ND	ND	11.15	ND	ND
Peanut oil 7	ND	ND	ND	61.76	ND	ND
Rapeseed oil1	ND	1528.26	ND	ND	ND	ND
Rapeseed oil2	ND	4782.28	ND	ND	ND	ND
Rapeseed oil3	ND	1383.87	ND	ND	ND	ND
Rapeseed oil4	ND	1586.55	ND	ND	ND	ND
Rapeseed oil5	ND	1716.62	ND	ND	ND	ND
Rapeseed oil6	ND	1920.35	ND	ND	ND	ND
Rapeseed oil7	ND	2063.55	ND	ND	ND	ND
Rapeseed oil8	ND	1716.52	ND	ND	ND	ND
Rapeseed oil9	ND	2097.09	ND	ND	ND	ND
Soybean oil1	2.58	20.10	6.15	ND	124.85	467.29
Soybean oil2	19.85	0.55	643.25	ND	16.92	13.81
Soybean oil3	1.09	0.48	160.01	ND	115.55	112.08
Soybean oil4	2.04	2.41	159.34	ND	44.15	27.40
Soybean oil5	9.43	1.61	824.62	ND	492.29	292.56
Soybean oil6	2.19	2.66	310.59	ND	258.66	181.66
Soybean oil7	9.71	1.88	328.49	ND	204.36	84.65
Soybean oil8	4.31	0.45	251.90	ND	159.23	99.18
Soybean oil9	4.60	1.58	409.18	ND	66.82	54.13
Soybean oil10	3.14	1.33	379.61	ND	61.59	37.51

Table S3. Concentration of the target markers after adulteration

Authentic oils	Compounds	Concentration of the target markers after adulteration						
		adulterated with 10% PO[a]	adulterated with 10% SO[b]	adulterated with 10% RO[c]	adulterated with 10% PO and 10% SO	adulterated with 10% PO and 10% RO	adulterated with 10% SO and 10% RO	adulterated with 10%SO,10% RO and 10%PO
camellia oil 3	Daidzin (ng/g)	ND	4.90 ± 0.19	ND	10.88 ± 0.00	ND	6.52 ± 0.05	11.10 ± 0.60
	Sinapic acid (ng/g)	ND	22.32 ± 0.12	239.46 ± 4.21	24.49 ± 0.56	250.74 ± 2.49	274.90 ± 6.96	252.35 ± 9.13
	Genistin (ng/g)	ND	17.47 ± 0.19	ND	33.25 ± 0.91	ND	19.66 ± 1.28	31.71 ± 0.72
	trans-Resveratrol (ng/g)	1.12 ± 0.52	ND	ND	2.05 ± 0.70	2.33 ± 0.12	ND	2.81 ± 0.14
	Daidzein (ng/g)	ND	8.18 ± 1.42	ND	9.70 ± 1.02	ND	13.13 ± 0.70	12.50 ± 0.74
	Genistein (ng/g)	ND	64.34 ± 0.90	ND	61.93 ± 4.13	ND	75.78 ± 2.53	76.98 ± 0.74
camellia oil 11	Daidzin (ng/g)	ND	7.20 ± 0.21	ND	6.45 ± 0.42	ND	6.05 ± 0.40	7.94 ± 0.36
	Sinapic acid (ng/g)	ND	28.10 ± 1.86	221.83 ± 3.13	26.39 ± 2.42	243.50 ± 5.12	254.85 ± 0.41	246.58 ± 0.35
	Genistin (ng/g)	ND	22.05 ± 0.57	ND	20.55 ± 0.58	ND	17.00 ± 0.77	22.94 ± 0.70
	trans-Resveratrol (ng/g)	0.76 ± 0.64	ND	ND	1.48 ± 0.29	3.08 ± 0.86	ND	1.67 ± 0.73
	Daidzein (ng/g)	ND	10.62 ± 0.65	ND	10.27 ± 0.32	ND	11.83 ± 1.04	12.07 ± 0.58
	Genistein (ng/g)	ND	70.21 ± 0.85	ND	67.26 ± 3.54	ND	71.25 ± 4.86	73.91 ± 2.54
camellia oil 12	Daidzin (ng/g)	ND	2.9 ± 0.12	ND	4.05 ± 0.29	ND	4.3 ± 0.08	4.76 ± 0.14
	Sinapic acid (ng/g)	ND	30.56 ± 1.66	229.92 ± 5.64	35.95 ± 2.49	241.62 ± 0.72	256.53 ± 12.05	253.51 ± 1.94
	Genistin (ng/g)	ND	8.29 ± 0.72	ND	13.12 ± 0.43	ND	13.49 ± 0.92	14.61 ± 0.16
	trans-Resveratrol (ng/g)	0.71 ± 0.65	ND	ND	3.08 ± 1.36	2.11 ± 0.18	ND	3.28 ± 1.12
	Daidzein (ng/g)	ND	9.15 ± 0.62	ND	11.69 ± 0.21	ND	10.82 ± 0.01	11.61 ± 0.08
	Genistein (ng/g)	ND	66.68 ± 1.39	ND	74.99 ± 1.83	ND	64.12 ± 2.27	69.19 ± 1.25

[a] PO = Peanut oil; [b] SO = Soybean oil; [c] RO = Rapeseed oil.
